# Supplementary material for: HIV-MTB Co-Infection Reduces CD4+ T Cells and Affects Granuloma Integrity
Source: Viruses. 2024 Aug 21;16(8):1335. doi: 10.3390/v16081335 (PMC11360352; doi:10.3390/v16081335)
Supplement: Supplementary file 1 [file viruses-16-01335-s001.zip › viruses-3094959-supplementary.pdf]

Table S1. The original data on the correlation of other immune cells and granuloma formation.

Subject No.	Granuloma formation	CD8+T cell count in peripheral blood (/mm^3)	Monocyte proportion in peripheral blood (%)	Neutrophils proportion in peripheral blood (%)
1	poorly formed	204	10.9	59.8
2	poorly formed	264	9	82.6
3	poorly formed	113	15.5	77.8
4	fully formed	548	8.1	77.4
5	poorly formed	548	8.1	77.4
6	poorly formed	191	6.1	79.3
7	poorly formed	867	5.8	86
8	poorly formed	211	15.4	62.9
9	poorly formed	419	11.4	48.2
10	poorly formed	263	19.1	45
11	poorly formed	111	4.6	85.9
12	poorly formed	148	10.5	81.4
13	poorly formed	141	13	64.8
14	poorly formed	408	12.2	70.6
15	poorly formed	808	5	81.8
16	poorly formed	367	8.1	49.6
17	poorly formed	453	21.3	62.9
18	poorly formed	516	13.8	72.4
19	poorly formed	147	6.8	83.3
20	poorly formed	561	6.8	72.4
21	poorly formed	440	9.2	72.6
22	fully formed	541	5.3	76.7
23	fully formed	216	5.9	67.1
24	poorly formed	537	7.8	84.8
25	fully formed	410	14.5	67.6
26	fully formed	338	6.7	78.5
27	poorly formed	564	25	42.9
28	poorly formed	755	10.5	64.1
29	poorly formed	607	9.6	73.4
30	poorly formed	269	7.2	79.7
31	poorly formed	831	6	81.4
32	poorly formed	319	16.9	45.3
33	fully formed	864	9.1	46.9
34	poorly formed	547	5.3	84.2
35	poorly formed	796	5	63
36	poorly formed	146	18.1	67.7
37	fully formed	280	5	89.7
38	fully formed	356	8.8	73.2
39	fully formed	551	0.61	62.5
40	poorly formed	1018	8.3	54
41	fully formed	859	10.5	29.8
42	poorly formed	750	20.1	49.8
43	fully formed	627	5.4	60.4
44	fully formed	236	9.7	60.3
45	poorly formed	244	10.8	59.5
46	poorly formed	-	6.5	72

47	poorly formed	-	7.1	53.2
48	poorly formed	-	3.7	89.8
49	poorly formed	-	4.8	80.2
50	poorly formed	-	10	66.9
51	poorly formed	-	-	-
52	fully formed	-	-	75.3
53	poorly formed	-	6.1	59.6

"-" means the subject did not have the results of the laboratory tests.
